# Comparison between Amnisure Placental Alpha Microglobulin-1 Rapid Immunoassay and Standard Diagnostic Methods for Detection of Rupture of Membranes

**DOI:** 10.1155/2013/587438

**Published:** 2013-09-01

**Authors:** Beng Kwang Ng, Pei Shan Lim, Mohamad Nasir Shafiee, Nur Azurah Abdul Ghani, Nor Azlin Mohamed Ismail, Mohd Hashim Omar, Muhammad Abdul Jamil Muhammad Yassin

**Affiliations:** Department of Obstetrics and Gynaecology, UKM Medical Centre, Malaysia

## Abstract

*Objective*. To determine the diagnostic accuracy of placental alpha microglobulin-1 assay and standard diagnostic methods for detecting rupture of membrane. *Study Design*. Prospective diagnostic study, between June 2011 to November 2011 at a tertiary centre. Initial evaluation included both the standard diagnostic methods for rupture of membranes and placental alpha microglobulin-1 immunoassay. The actual rupture of membranes was diagnosed on review of the medical records after delivery (absence of membrane or a positive pad chart). *Main Outcome Measures*. Placental alpha microglobulin-1 immunoassay and standard diagnostic methods for diagnosis of rupture of membrane. *Results*. A total of 211 patients were recruited. At initial presentation, 187 patients (88.6%) had ruptured membranes, while 24 patients (11.4%) had intact membranes. Placental alpha microglobulin-1 immunoassay confirmed rupture of membranes at initial presentation with a sensitivity of 95.7% (179 of 187), specificity of 100% (24 of 24), positive predictive value of 100% (179 of 179), and negative predictive value of 75.0% (24 of 32). By comparison, the conventional standard diagnostic methods had a sensitivity of 78.1% (146 of 187), specificity of 100% (24 of 24), positive predictive value of 100% (146 of 146), and negative predictive value of 36.9% (24 of 65) in diagnosing rupture of membrane. *Conclusion*. Placental alpha-microglobulin-1 immunoassay is a rapid and accurate method for confirming the diagnosis of rupture of membrane. It was superior to conventional standard diagnostic methods (pooling, nitrazine, and ferning), the nitrazine test alone or fern test alone.

## 1. Introduction

Prelabour rupture of the membranes (PROM) is defined as rupture of the fetal membranes before the onset of labour. It complicates about 10% of pregnancies [1]. Preterm PROM (PPROM) contributes for 20%–40% of PROM [2]. It is associated with significant perinatal morbidity which includes preterm delivery, lungs hypoplasia, fetal deformities, and postnatal endometritis [1].

An accurate diagnosis with prompt and correct intervention is of utmost importance in the management of ROM especially in preterm gestation. Clinicians have to weigh between risk of prolonging the pregnancy and risk of neonatal morbidity from abruptio placenta, chorioamnionitis and preterm delivery [3–5]. Failure to ascertain the correct diagnosis would result in either failure to initiate proper treatment or would lead to unnecessary interventions such as hospitalization and inappropriate use of antibiotics and antenatal corticosteroids, as well as induction of labour [6–8]. This will lead to increased maternal and fetal morbidity and mortality [2] which in turn leads to higher health care cost [9].

The accurate diagnosis of rupture of membranes can be difficult in obstetrics practice. The use of indigo carmine injection remains the diagnostic gold standard. However, it is too invasive to be used as routine practice. An ideal diagnostic tool should be noninvasive, able to detect ROM (sensitivity), exclude subclinical ROM (specificity), differentiate between amniotic fluid and other physiological fluids (cervicovaginal secretion, blood, and semen), and provide a rapid result (bedside test). Unfortunately, this test is not available currently.

With the exception of amniotic fluid being visualised directly from the cervical os, each of the available conventional standard diagnostic methods for diagnosing ROM has its own limitation [10–17]. Nitrazine test is to detect an alkaline pH in the amniotic fluid. Unfortunately, it has a high false-positive rate as vaginitis, cervicitis, urine, blood, and semen or antiseptic agents may give rise to an alkaline pH [11, 12]. The reported sensitivity of nitrazine test ranged from 90% to 97% with specificity from 16% to 70% [13, 14].

Fern test gives a sensitivity and specificity of 51% and 70%, respectively, when patients were not in labour and increased to 98% and 88%, respectively, when used in patients in labour [17]. Visualisation of crystal of amniotic fluid on the slide may give false-positive result in the presence of semen and cervical mucus. On the other hand, contamination with blood or a dry swab as a result of technical error may lead to false-negative results [11, 15, 16].

Absence of an accurate noninvasive diagnostic test for ROM result in the emergence of various commercial tests using biochemical markers as indicator. For example, fetal fibronectin, actimPROM (insulin-like growth factor binding protein-1 immunoassay), alpha-fetoprotein, and vaginal prolactin. Most of these biochemical markers failed to achieve an acceptable accuracy that is required in an ideal gold standard diagnostic test [18–21].

Recently, a bedside immunoassay (AmniSure rapid immunoassay) has been used to detect fetal glycoprotein, placental alpha microglobulin-1(PAMG-1), and in cervicovaginal secretions [20]. Placental alpha microglobulin-1 is considered an ideal substance to be used for detection of ROM. It has a concentration from 1,000- to 10,000-fold higher in amniotic fluid than in the cervicovaginal secretion (2,000–25,000 ng/mL versus 0.05–2.0 ng/mL) [20, 21]. There is currently limited data available on the use of of PAMG-1 immunoassay in clinical practice.

This study is to compare the diagnostic accuracy of this immunoassay with that of conventional standard diagnostic methods in diagnosing ROM. The standard diagnostic method of diagnosing rupture of membrane is defined as positive for two of the following three clinical signs [22]:

1. visualisation of fluid pooling in the posterior fornix,
2. positive ferning test,
3. positive nitrazine test.

## 2. Materials and Methods

### 2.1. Study Design

This was a prospective diagnostic study between June 2011 and November 2011 in a tertiary teaching hospital. Pregnant women who presented with symptoms of ROM either in labour or not in labour, gestational age from 24 weeks onwards, and those consented to the study were recruited. We excluded pregnant women with active vaginal bleeding, those diagnosed to have placenta praevia and those with intrauterine death.

### 2.2. Procedure

All eligible pregnant women were informed regarding the study, and they were provided with a patient information sheet. A written consent was obtained. A detailed history, physical examination, sterile speculum examination, and transabdominal ultrasound examination were performed. All participants were assessed using the PAMG-1 rapid immunoassay test (AmniSure), nitrazine test (Amnicator), and ferning test at the initial speculum examination.

A positive fern test is defined as visualisation of arborisation or crystallization of amniotic fluid observed microscopically. At sterile speculum examination, after visualisation for any presence of pooling of liquor, swab from posterior vaginal fornix or pooling site was performed with a sterile Dacron swab for fern test. The swab was smeared against a glass slide to create a very thin smear. The slide was then allowed to dry under room air for about 10 minutes without any heating. Finally, the slide was examined under a microscope for arborisation. Care was taken not to contaminate the slide with fingerprint, and technical errors such as dry swab were avoided. These steps were taken to reduce the false-positive and false-negative results in fern test. To reduce interpersonal data interpretation error, this test was performed by single operator. 

Nitrazine test (Amnicator) and PAMG-1 (AmniSure) rapid immunoassay test were performed at the same time. The Amnicator stick was directed to the pooling of fluid at the posterior vaginal fornix. If there was no pooling of fluid seen, then the Amnicator would be directed to swab the posterior fornix. A positive result was interpreted as a change of the Amnicator stick from orange to dark blue colour. 

PAMG-1 rapid immunoassay test (AmniSure) was performed by placing a sterile Dacron swab at the posterior vaginal fornix for about one minute. It was then soaked into a vial containing solvent for another 1 minute. The test strip was then put in the solvent, and the result would be available in 5 minutes. 

Results of the above tests were recorded. The result of pooling of liquor and Amnicator test would be noted to the attending clinician who then decides on further management of the patient according to the hospital protocol. However, results of fern test and PAMG-1 would be blinded to the attending clinician as this is not a routine hospital practice. Patient's records were reviewed after delivery. Data regarding the outcome of the pregnancy such as induction of labour or evidence of chorioamnionitis and outcome of the fetus (admission to NICU, pulmonary hypoplasia, fetal deformities, respiratory distress syndrome, and infection morbidity) were collected for analysis. For the purposes of this study, actual ROM was considered when the membranes are absent during vaginal examination or a positive pad chart was obtained.

### 2.3. Statistical Analysis

All data in the checklist was collected in an electronic database and analysed using SPSS Version 16.0. The nonnormally distributed variables were evaluated with nonparametric test. 

## 3. Result

**Figure 1:**
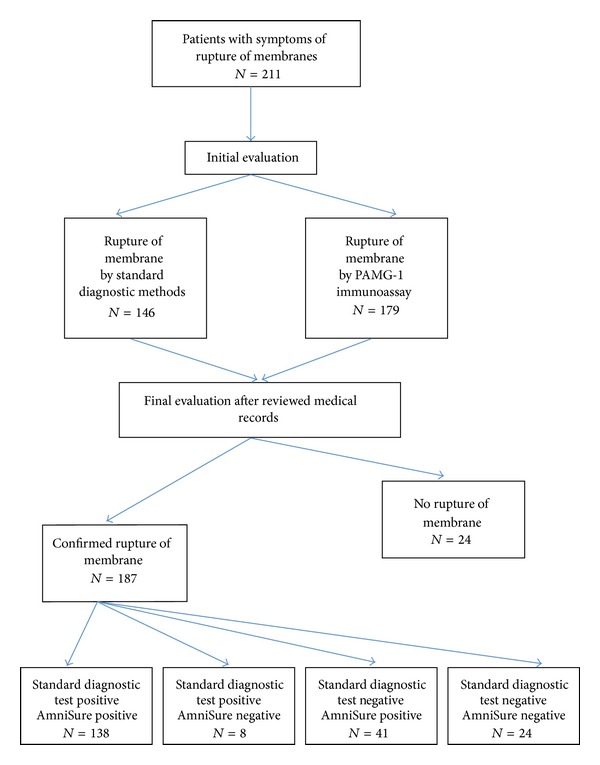
Summary of results for both standard diagnostic method and AmniSure.

A total of 211 pregnant women were eligible and consented to the study. The demographic data and clinical findings on admission were shown in the Table 1. Pooling of liquor was seen in 76.8%, 55.5% positive fern test, 68.7% positive nitrazine test, and 84.8% positive PAMG-1. From the total 211 patients, diagnosis of ROM was initially made in 69.2% (146/211) using conventional standard diagnostic methods and 84.8% (179/211) using the PAMG-1 immunoassay (Figure 1). Subsequent review of the case notes confirmed that 187 out of 211 patients (88.6%) had actual ruptured membranes, whereas 24 patients (11.4%) had intact membranes. 

Using the final determination as the confirmation of ROM, PAMG-1 immunoassay had sensitivity of 95.7% (179/187), specificity of 100% (24/24), positive predictive value (PPV) of 100% (179/179), and negative predictive value (NPV) of 75.0% (24/32) for confirmation of ROM at initial presentation. In contrast, the conventional standard diagnostic methods (pooling, nitrazine, and ferning) confirmed ROM with a sensitivity of 78.1% (146/187), specificity of 100% (24/24), PPV of 100% (146/146), and NPV of 36.9% (24/65). The PAMG-1 immunoassay was a statistically significant better test in detecting ROM compared to the conventional standard diagnostic methods (95.7% [179/187] versus 78.1% [146/187], *P* < 0.000, McNemar test) (Table 2). Both the conventional standard diagnostic methods and PAMG-1 immunoassay had an excellent specificity (100%). 

**Figure 2:**
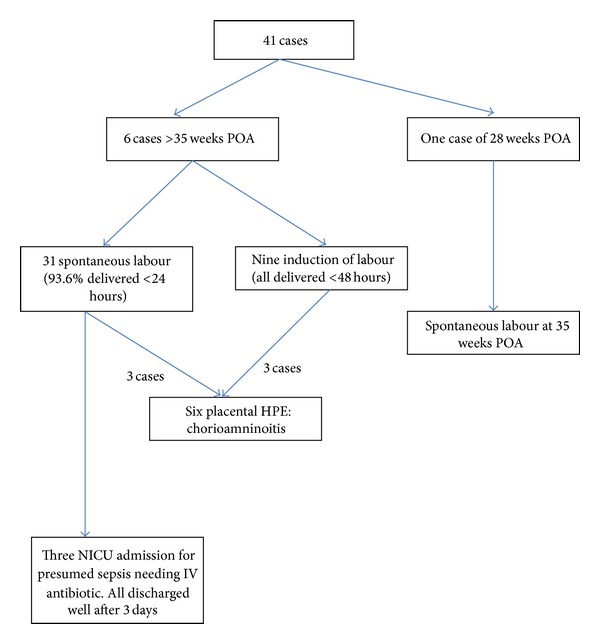
Positive AmniSure with negative standard diagnostic method in actual ROM.

**Figure 3:**
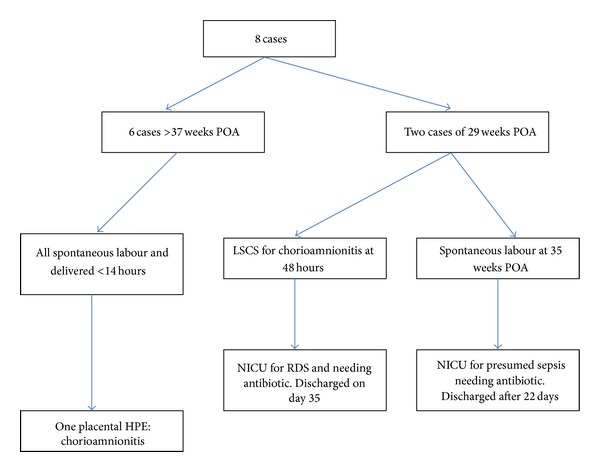
Negative AmniSure with positive standard diagnostic method in actual ROM.

There were 49 cases where the results of conventional standard diagnostic methods were in disagreement with PAMG-1 immunoassay. All the 41 patients with a positive PAMG-1 immunoassay result but a negative standard diagnostic methods were subsequently confirmed having ROM based on absence of membrane and/or positive pad charting in the ward. Their delivery outcomes were shown in the Figure 2. Eight patients here were having negative AmniSure but positive standard diagnostic method although seven of them had pooling of liquor seen upon speculum examination. Their delivery outcomes were summarised in Figure 3.

When comparing to nitrazine test as well as fern test, the sensitivity of PAMG-1 immunoassay was significantly higher (97.5% [179/187] versus 77.5% [145/187] and 62.6% [117/187]). All the above tests have a 100% specificity and positive predictive value. However, the negative predictive value is low for standard diagnostic methods: nitrazine test, fern test, and pooling of liquor with value ranging from as low as 25.5 to 49.0. Where else, PAMG-1 immunoassay gave a reasonably acceptable negative predictive value of 75.0.

## 4. Discussion

Preterm PROM is associated with significant maternal and perinatal mortality and morbidity [3, 22, 24, 25]. Unfortunately, there is absence of an accurate and simple diagnostic tool to establish the diagnosis [6, 10] as the traditional way to diagnose ROM is subjective. The traditional “gold standard” relied heavily on the ability of the attending healthcare personnel to visualise pooling of liquor in the posterior vaginal fornix, detecting an alkaline vaginal pH, and observation of ferning effect from the liquor. However, each of these standard diagnostic methods was associated with high false-positive or -negative results [10, 12].

In the present study, the PMAG-1 immunoassay alone has an overall sensitivity of 97.5%, specificity of 100%, PPV of 100%, and NPV of 75.0%. Hence, it is superior to nitrazine test or fern test alone as well as in combination (conventional standard diagnostic methods) (Table 2). It was also found to be accurate in the majority of women (95.7%) that confirmed to have actual ROM later even though standard diagnostic methods were negative in some of them. 

Several biochemical markers have been studied—including fetal fibronectin, [20] alpha-fetoprotein, [26] and insulin-like growth factor binding protein-1 [12, 13, 26]—to improve the accuracy of ROM detection. However, none had shown a promising result. The AmniSure immunoassay involves detecting the presence of high concentration of glycoprotein PAMG-1 in the cervicovaginal secretion. PAMG-1 is a 34-kd glycoprotein [27]. Due to its unique features (i.e., high concentration in the amniotic fluid, low concentration in blood, and very low concentration in cervicovaginal discharge), it was suitable to be used as marker for ROM. In order to minimise false positive or negative, two monoclonal antibodies were used to determine the sensitivity threshold level of AmniSure. The lowest level of PAMG-1 measured by this monoclonal antibodies was 0.05 to 0.2 ng/mL from the cervicovaginal secretion. PAMG-1 found in amniotic fluid was at the range from 2,000 to 25,000 ng/mL. When there was rupture of membrane, the level of PAMG-1 increased significantly in vaginal secretion. With its optimal sensitivity threshold at 5 ng/mL, AmniSure reduces the chance of faulty results. 

The PAMG-1 immunoassay provides a quality diagnostic tool that was rapid, accurate, and with higher sensitivity and specificity compared to the other methods used currently. First of all, AmniSure can be done without inserting a speculum with what is being practised currently. It also serves as single test that is able to help determining and establishing the correct diagnosis, especially when the diagnosis of ROM is inconclusive.

The current available data on the clinical PAMG-1 immunoassay is sparce despite theoretical benefits of PAMG-1 in detecting ROM. Cousins et al. [21] had compared the use of PAMG-1 with standard diagnostic methods for diagnosing ROM. He concluded that the PAMG-1 immunoassay was more superior to establish the correct diagnosis of ROM compared with other conventional diagnostic test. The present study was largely in agreement with Cousins et al. and other trials except the negative predictive value from our study which is lower than other studies (75.0 versus 91.3–99.1). (Table 3) [21, 23] We demonstrated that the PAMG-1 immunoassay was superior to the conventional standard diagnostic methods, nitrazine test or fern test alone. In all 41 cases (100%), where there was a discrepancy results between the PAMG-1 immunoassay (positive) and conventional standard diagnostic methods (negative), the PAMG-1 immunoassay was found to be more accurate. This might be due to the fact that it was more sensitive to detect subclinical ROM from microperforations. Unfortunately, its usage is currently limited due to the cost factor. However, in cases where the diagnosis is in doubt, the placental alpha microglobulin-1 immunoassay is a valuable tool to diagnose rupture of membranes. There were 8 patients in which the PAMG-1 results were negative, but the standard diagnostic method yielded a positive result and the outcomes of pregnancy were also summarized in Figure 3. Cousins et al. [21], in their his study noted 5 false negative results with AmniSure, but after retesting, 4 gave true positive results with one true negative result. The author suspected that this was due to defective test kit. However, in our study, a repeat AmniSure was not done for the 8 patients with false negative results. These false negative results could be due to sampling failure despite obvious pooling of liquor was seen in majority of them. When calculating the kappa to test the agreement between both AmniSure and standard diagnostic method, it was noted that the number of observed agreement was 162 (76.78%) and the number of agreements expected by chance was 133.7 (67.37%). Thus, the kappa obtained was 0.366 (95% confidence interval 0.232–0.500). Therefore, the strength of agreement was considered to be fair. 

## 5. Limitation 

The gold standard to diagnose rupture of membrane is injection of indigo carmine directly into the amniotic sac. However, it is too invasive to be used as routine practice or for research purposes. As a result, we could only rely on noninvasive clinical features such as absence of membranes and positive pad chart as clinical guide for confirming leaking liquor. 

## 6. Conclusion

The placental alpha microglobulin-1 immunoassay is noninvasive, rapid, and accurate in detecting ROM. Its performance appeared to be superior compared to conventional standard diagnostic tests (pooling, nitrazine, and ferning), nitrazine test, or fern test alone. The use of AmniSure as a diagnostic tool helps to establish the correct diagnosis and determine the treatment options. Unfortunately, its usage is currently limited by its cost. In cases where the diagnosis is in doubt particularly in preterm prelabour rupture of membranes, the placental alpha microglobulin-1 might act as a valuable tool.

## Figures and Tables

**Table 1:** Demographic data and clinical findings on admission.

	Total *N* = 211
Age (years)	28.0 (26.0, 28.0)
Ethnic *n* (%)	
Malay	166 (78.7)
Chinese	27 (12.8)
Indian	12 (5.7)
Others	6 (2.8)
Parity	0 (0, 1)
Gestation age (weeks) at presentation	38.3 (36.5, 39.3)
Gestational age <34 weeks *n* (%)	19 (9.0)
History	
Duration of rupture membrane (hours)	1.5 (1.0, 4.0)
Presence of contraction	134 (63.5)
Presence of fever	3 (1.4)
Presence of vaginal bleeding	0
Presence of vaginal discharge	11 (5.2)
Examination	
Maternal tachycardia	29 (13.7)
Abdominal tenderness	1 (0.5)
Pooling of liquor	162 (76.8)
Os dilatation (cm)	2 (1, 3)

All quantitative data were presented in median (quartile) unless specified.

**Table 2:** Final determination of rupture of membrane and performance metrix of each test.

Test	Result		Sensitivity (%)	Specificity (%)	Positive predictive value (%)	Negative predictive value (%)
	Positive	Negative				
Standard diagnostic methods	146	41	78.1	100	100	36.9
Placenta alpha microglobulin-1	179	8	95.7	100	100	75.0
Nitrazine test	145	42	77.5	100	100	36.3
Fern test	117	70	62.6	100	100	25.5
Pooling of liquor	162	25	86.6	100	100	49.0

**Table 3:** Comparison to other trials.

Study	Number of patients	Sensitivity (%)	Specificity (%)	Positive predictive value (%)	Negative predictive value (%)
Cousins et al. [21]	203	98.9	100	100	99.1
Lee et al. [23]	184	98.7	87.5	98.1	91.3
Our study	211	95.7	100	100	75.0
